# Glucose-6-Phosphate Dehydrogenase Status and Risk of Hemolysis in Plasmodium falciparum-Infected African Children Receiving Single-Dose Primaquine

**DOI:** 10.1128/AAC.02889-14

**Published:** 2014-08

**Authors:** Alice C. Eziefula, Helmi Pett, Lynn Grignard, Salome Opus, Moses Kiggundu, Moses R. Kamya, Shunmay Yeung, Sarah G. Staedke, Teun Bousema, Chris Drakeley

**Affiliations:** a Faculty of Infectious and Tropical Diseases, London School of Hygiene and Tropical Medicine, London, United Kingdom; b Department of Medical Microbiology, Radboud University Medical Center, Nijmegen, The Netherlands; c Infectious Diseases Research Collaboration, Kampala, Uganda; d Department of Medicine, Makerere University College of Health Sciences, Kampala, Uganda

## Abstract

Glucose-6-phosphate dehydrogenase (G6PD) enzyme function and genotype were determined in Ugandan children with uncomplicated falciparum malaria enrolled in a primaquine trial after exclusion of severe G6PD deficiency by fluorescent spot test. G6PD A− heterozygotes and hemizygotes/homozygotes experienced dose-dependent lower hemoglobin concentrations after treatment. No severe anemia was observed.

## TEXT

Declines in malaria due to Plasmodium falciparum have been documented in a number of settings where malaria is endemic. It is debated whether scaling-up of conventional malaria control will sustain these declines or achieve elimination unless augmented by tools that specifically reduce transmission. Primaquine is the only currently available drug that actively clears mature P. falciparum gametocytes and prevents malaria transmission to mosquitoes (1). The wide-scale use of primaquine is hampered by its hemolytic effect in people with glucose-6-phosphate dehydrogenase (G6PD) deficiency. The mutation deficiency alters G6PD enzyme function (2), exposing red blood cells to oxidative stress and resultant hemolysis in the presence of a stressor, such as primaquine (3, 4). Primaquine-induced hemolysis is dose related (1, 5, 6). While testing for G6PD deficiency is widely recommended prior to the radical treatment of Plasmodium vivax with 14 days of primaquine, P. falciparum transmission may be considerably reduced by a single, low dose of primaquine (1, 7) and may avoid the necessity to screen for G6PD deficiency. We determined G6PD enzyme function and the presence of the most common African G6PD mutation (G6PD A−; 202A/376G) in a cohort of Ugandan children treated with low-dose primaquine for clearing P. falciparum gametocytes. This was a randomized, double-blinded placebo controlled trial with four parallel arms. Ugandan children 1 to 10 years old with uncomplicated P. falciparum malaria, hemoglobin concentration (Hb) of ≥8 g/dl, and normal G6PD enzyme function based on a fluorescent spot test (FST; R&D Diagnostics, Agia Paraskevi, Greece) were enrolled and randomized to treatment with artemether lumefantrine (AL) alone or with a single dose of primaquine at 0.1, 0.4, or 0.75 mg/kg of body weight on the last day of AL treatment (7, 8). Genotyping of G6PD 202A and G6PD 376G was performed (9, 10). Hb was measured on days 0, 1, 2, 3, 7, 10, 14, 21, and 28 after enrollment by HemoCue 201+ (Angelholm, Sweden) and expressed as absolute and relative change compared to baseline values. These values were normally distributed, presented using mean values and standard deviations, and analyzed using linear regression models. Because the age distribution of the red blood cell population influences the severity of drug-induced hemolysis (11), we adjusted all comparisons for baseline Hb concentration. All trial participants (*n* = 468) were G6PD normal by FST. DNA was available for 461 individuals of whom 27 (5.9%) were homozygous/hemizygous, 61 were heterozygous (13.2%), and 373 (80.9%) were normal for the G6PD variant A− (wild type [WT]). All individuals with the 202A mutation also had the 376G mutation, and individuals were classified based on the 202A mutation (Table 1). G6PD 202 A− heterozygous individuals experienced a mean reduction in Hb concentration on day 7 after treatment of 1.08 g/dl (standard deviation [SD], 1.14; *P* = 0.048) in the 0.75-mg/kg treatment arm and 0.99 g/dl (SD, 1.48; *P* = 0.054) in the 0.4-mg/kg treatment arm (Table 2). Homozygous/hemizygous individuals in the 0.75-mg/kg and 0.4-mg/kg arms also experienced a reduction in absolute Hb concentration on day 7, although this was statistically significant in the 0.4-mg/kg arm only (*P* = 0.043). When changes in Hb concentration on day 7 were expressed as a proportion of baseline Hb concentration, the same trend was observed with statistically significant decreases in the 0.75-mg/kg arm for heterozygous individuals and in the 0.4-mg/kg arm for homozygous/hemizygous individuals. No statistically significant changes in absolute or relative Hb concentrations were observed for heterozygous or homozygous/hemizygous individuals in the 0.1-mg/kg arm or placebo arm (Table 2). We found no explanation for the numerically large, but statistically nonsignificant, reduction in Hb concentration in homozygous/hemizygous individuals on day 7 after receiving AL without primaquine. A previous study found no hemolysis after AL in homozygous/hemizygous individuals (12), and we conclude our observation may be a spurious finding and related to our small sample size. We observed no statistically significant associations between G6PD genotype and absolute or relative Hb concentrations in any treatment arm on days 3 and 10 after initiation of treatment (see the supplemental material). Sixty-nine individuals experienced a reduction of >2 g/dl in the first 2 weeks of follow-up: 13.7% (51/373) of the WT individuals, 26.2% (16/61; *P* = 0.031) of the heterozygous individuals, and 7.4% (2/27; *P* = 0.48) of the G6PD 202 A− homozygous/hemizygous individuals (*P* = 0.020). For all individuals, Hb concentrations normalized during follow-up. The current findings provide important data on the hemolytic effect of single-, low-dose primaquine. Our results show that the predominant test for G6PD deficiency screening, the FST (13), failed to identify a substantial proportion of individuals who were genotypically G6PD deficient, particularly female heterozygotes, who experienced significant reductions in hemoglobin following higher doses of primaquine. The observation that some G6PD-deficient individuals were FST normal is unsurprising since the test may be insufficiently sensitive to detect mild G6PD deficiency (13), but there are few supportive published data. We observed statistically significant decreases in Hb following single-dose primaquine in these G6PD-deficient individuals. A hemolytic effect of a single dose of 0.75 mg/kg primaquine base has been reported before (6); our study shows that a reduction in Hb concentrations is also evident after a single dose of 0.4 mg/kg but not 0.1 mg/kg. Moreover, reductions in Hb were transient, with no participant experiencing clinical symptoms suggestive of anemia and none requiring related clinical care. Although these findings are notable, a major limitation of the study is that individuals who were determined G6PD deficient based on the FST were excluded from the study (*n* = 32), thereby plausibly removing those most severely deficient and thereby those with the highest risk of primaquine-induced hemolysis. There is therefore a need for confirmatory trials to formally assess primaquine safety in G6PD-deficient individuals, in particular with the World Health Organization recommended dose of 0.25 mg/kg. Such studies will have to take into account interindividual differences in primaquine metabolism that determine primaquine efficacy in P. vivax (14) and potentially also safety.

**TABLE 1:** Baseline characteristics

Characteristic	Value by G6PD 202 A− genotype				
	Wild type	Heterozygous	*P* value for difference from wild type	Homozygous/hemizygous	*P* value for difference from wild type
No. of participants (% study population)	373 (80.9)	61 (13.2)		27 (5.9)	
% female (no. of females/total no. of participants)	46.7 (174/373)	100.0 (61/61)	<0.001	3.7 (1/27)	<0.001
Mean (SD) age in yrs	5.0 (2.6)	4.8 (2.3)	0.61	4.9 (2.4)	0.86
Mean (SD) baseline Hb concn in g/dl	11.2 (1.5)	11.4 (1.4)	0.20	10.9 (1.4)	0.38
% 376G genotype (no. of participants with genotype/total no.)					
Heterozygous	18.6 (69/371)	78.7 (48/61)		0.0 (0/27)	
Homozygous	12.9 (48/371)	21.3 (13/61)	<0.001	100.0 (27/27)	<0.001

**TABLE 2:** G6PD 202 A− genotype and hemoglobin levels^*a*^

Characteristic	Value by treatment arm			
	0.75 mg/kg primaquine	0.4 mg/kg primaquine	0.1 mg/kg primaquine	Placebo
No. of study participants				
G6PD normal	98	90	93	92
G6PD heterozygous	14	13	16	18
G6PD hemizygous/homozygous	4	10	6	7
Mean absolute change (SD) in Hb on day 7				
G6PD normal, in g/dl	−0.41 (0.95)	−0.25 (1.22)	−0.30 (1.07)	−0.11 (1.33)
G6PD heterozygous, in g/dl	−1.08 (1.14)	−0.99 (1.48)	−0.07 (0.98)	−0.49 (1.40)
*P* value	0.048	0.054	0.35	0.28
G6PD hemizygous/homozygous, in g/liter	−1.10 (1.34)	−0.48 (0.76)	−0.07 (1.21)	−1.02 (0.81)
*P* value	0.21	0.043	0.91	0.22
% relative change (SD) in Hb on day 7, in g/dl				
G6PD normal	−3.25 (8.60)	−1.28 (11.24)	−2.16 (9.71)	−0.23 (11.34)
G6PD heterozygous	−9.38 (10.4)	−7.79 (12.57)	−0.01 (8.56)	−3.26 (12.26)
*P* value	0.044	0.073	0.34	0.33
G6PD hemizygous/homozygous	−7.97 (12.40)	−4.29 (7.70)	−0.05 (11.45)	−8.59 (7.28)
*P* value	0.36	0.028	0.93	0.16

a On day 7 after initiation of treatment with artemether-lumefantrine (AL) plus placebo or AL plus different doses of primaquine. All primaquine or placebo treatment was administered together with six doses of AL; primaquine/placebo was given on day 2 of treatment, together with dose 5 of AL. *P* values are compared to G6PD-normal individuals, adjusted for baseline Hb concentration.

## Supplementary Material

Supplemental material
