# Supplementary material for: Glucose-6-Phosphate Dehydrogenase Status and Risk of Hemolysis in Plasmodium falciparum-Infected African Children Receiving Single-Dose Primaquine
Source: Antimicrob Agents Chemother. 2014 Aug;58(8):4971–3. doi: 10.1128/AAC.02889-14 (PMC4136063; doi:10.1128/AAC.02889-14)
Supplement: Supplemental material [file supp_58_8_4971__index.html]

Glucose-6-Phosphate Dehydrogenase Status and Risk of Hemolysis in Plasmodium falciparum-Infected African Children Receiving Single-Dose Primaquine — Supplemental material 

# Glucose-6-Phosphate Dehydrogenase Status and Risk of Hemolysis in Plasmodium falciparum-Infected African Children Receiving Single-Dose Primaquine

## Supplemental material

**Files in this Data Supplement:**

- Supplemental file 1 -

  Supplemental Tables S1 to S3.

  PDF, 187K
