# Supplementary material for: Glucose-6-Phosphate Dehydrogenase Status and Risk of Hemolysis in Plasmodium falciparum-Infected African Children Receiving Single-Dose Primaquine
Source: Antimicrob Agents Chemother. 2014 Aug;58(8):4971–3. doi: 10.1128/AAC.02889-14 (PMC4136063; doi:10.1128/AAC.02889-14)
Supplement: Supplemental material [file AAC.02889-14_zac008143166so1.pdf]

Table S1. G6PD 202 A- genotype and haemoglobin levels on day 3 after initiation of treatment with Artemether-Lumefantrine (AL) plus placebo or AL plus different doses of primaquine

**Treatment arm**		**0.75 mg/kg primaquine**	**0.4 mg/kg primaquine**	**0.1 mg/kg primaquine**	**placebo**
**Absolute change in Hb concentration on day 3 compared to baseline**					
G6PD normal	mean change, g/dL (sd)	-0.71 (0.85)	-0.47 (0.97)	-0.63 (0.96)	-0.46 (1.17)
G6PD heterozygous	mean change, g/dL (sd)	-1.25 (1.13)	-0.96 (0.80)	-0.66 (1.08)	-1.05 (1.09)
	Comparison G6PD normal, β (se); p-value*	-0.45 (0.25); 0.07	-0.36 (0.25); 0.14	-0.02 (0.25); 0.95	-0.48 (0.26); 0.066
G6PD hemi-/homozygous	mean change, g/dL (sd)	-0.65 (0.66)	-0.29 (1.05)	-0.55 (1.06)	-1.12 (0.94)
	Comparison G6PD normal, β (se) p-value*	0.12 (0.44); 0.78	-0.08 (0.28); 0.78	-0.11 (0.39); 0.78	-0.39 (0.42); 0.35
**Relative change in Hb on day 3 as percentage of baseline concentration**					
G6PD normal	% change (sd)	-6.26 (7.66)	-3.64 (8.63)	-5.46 (8.56)	-3.49 (9.79)
G6PD heterozygous	% change (sd)	-10.75 (10.15)	-7.95 (6.80)	-5.53 (9.59)	-8.37 (8.80)
	Comparison G6PD normal, β (se); p-value*	-3.96 (2.27); 0.083	-3.30 (2.30); 0.15	-0.004 (2.30) 1.00	-4.12 (2.27); 0.073
G6PD hemi-/homozygous	% change (sd)	-4.86 (5.82)	-2.45 (10.03)	-4.92 (9.75)	-9.48 (8.47)
	Comparison G6PD normal, β (se) p-value*	1.76 (4.02); 0.66	-0.86 (2.60); 0.74	-0.79 (3.60); 0.83	-4.07 (3.63) 0.27

All primaquine or placebo treatment was administered together with six doses of artemether-lumefantrine (AL); primaquine/placebo was given on day 2 of treatment, together with dose 5 of AL. Sd=standard deviation; * p-value compared to G6PD normal individuals, adjusted for baseline Hb concentration.

Table S2. G6PD 202 A- genotype and haemoglobin levels on day 7 after initiation of treatment with Artemether-Lumefantrine (AL) plus placebo or AL plus different doses of primaquine

**Treatment arm**		**0.75 mg/kg primaquine**	**0.4 mg/kg primaquine**	**0.1 mg/kg primaquine**	**placebo**
**Absolute change in Hb concentration on day 7 compared to baseline**					
G6PD normal	mean change, g/dL (sd)	-0.41 (0.95)	-0.25 (1.22)	-0.30 (1.07)	-0.11 (1.33)
G6PD heterozygous	mean change, g/dL (sd)	-1.08 (1.14)	-0.99 (1.48)	-0.07 (0.98)	-0.49 (1.40)
	Comparison G6PD normal, β (se); p-value*	-0.52 (0.26); 0.048	-0.55 (0.28); 0.054	0.24 (0.26); 0.35	-0.30 (0.28); 0.28
G6PD hemi-/homozygous	mean change, g/dL (sd)	-1.10 (1.34)	-0.48 (0.76)	-0.07 (1.21)	-1.02 (0.81)
	Comparison G6PD normal, β (se) p-value*	-0.59 (0.47); 0.21	-0.66 (0.32); 0.043	-0.05 (0.40); 0.91	-0.57 (0.46); 0.22
**Relative change in Hb on day 7, as percentage of baseline concentration**					
G6PD normal	% change (sd)	-3.25 (8.60)	-1.28 (11.24)	-2.16 (9.71)	-0.23 (11.34)
G6PD heterozygous	% change (sd)	-9.38 (10.4)	-7.79 (12.57)	-0.01 (8.56)	-3.26 (12.26)
	Comparison G6PD normal, β (se); p-value*	-4.93 (2.42); 0.044	-4.82 (2.66); 0.073	2.28 (2.35); 0.34	-2.43 (2.47); 0.33
G6PD hemi-/homozygous	% change (sd)	-7.97 (12.40)	-4.29 (7.70)	-0.05 (11.45)	-8.59 (7.28)
	Comparison G6PD normal, β (se) p-value*	-3.91 (4.29); 0.36	-6.71 (3.02); 0.028	-0.33 (3.68); 0.93	-5.66 (4.04); 0.16

All primaquine or placebo treatment was administered together with six doses of artemether-lumefantrine (AL); primaquine/placebo was given on day 2 of treatment, together with dose 5 of AL. Sd=standard deviation; * p-value compared to G6PD normal individuals, adjusted for baseline Hb concentration.

Table S3. G6PD 202 A- genotype and haemoglobin levels on day 10 after initiation of treatment with Artemether-Lumefantrine (AL) plus placebo or AL plus different doses of primaquine

**Treatment arm**		**0.75 mg/kg primaquine**	**0.4 mg/kg primaquine**	**0.1 mg/kg primaquine**	**placebo**
**Absolute change in Hb on day 10**					
G6PD normal	mean g/dL (sd)	-0.13 (1.14)	0.07 (1.22)	-0.16 (1.24)	-0.06 (1.21)
G6PD heterozygous	mean g/dL (sd)	-0.87 (0.82)	-0.35 (1.46)	0.17 (0.98)	0.01 (1.32)
	Comparison G6PD normal, β (se); p-value*	-0.54 (0.28); 0.06	-0.19 (0.29); 0.52	0.35 (0.27); 0.19	0.02 (0.25); 0.93
G6PD hemi-/homozygous	mean g/dL (sd)	-0.80 (1.36)	0.10 (0.99)	0.43 (1.14)	-0.68 (0.84)
	Comparison G6PD normal, β (se) p-value*	-0.53 (0.50); 0.29	-0.37 (0.33); 0.26	0.20 (0.42); 0.63	-0.42 (0.40); 0.31
**Relative change in Hb on day 10**					
G6PD normal	% change g/dL (sd)	-0.51 (10.76)	1.49 (11.77)	-0.49 (11.76)	1.37 (10.64)
G6PD heterozygous	% change g/dL (sd)	-7.53 (7.26)	-1.93 (12.54)	1.99 (9.01)	1.30 (12.27)
	Comparison G6PD normal, β (se); p-value*	-5.13 (2.69); 0.059	-1.34 (2.82); 0.64	2.69 (2.53); 0.29	0.56 (2.22); 0.80
G6PD hemi-/homozygous	% change g/dL (sd)	-5.16 (13.39)	1.48 (10.11)	5.06 (11.46)	-5.60 (7.13)
	Comparison G6PD normal, β (se) p-value*	-3.34 (4.76); 0.48	-3.67 (3.19); 0.25	1.80 (3.96); 0.65	-4.12 (3.64); 0.26

All primaquine or placebo treatment was administered together with six doses of artemether-lumefantrine (AL); primaquine/placebo was given on day 2 of treatment, together with dose 5 of AL. Sd=standard deviation; * p-value compared to G6PD normal individuals, adjusted for baseline Hb concentration.
